# Bipolar Androgen Therapy: When Excess Fuel Extinguishes the Fire

**DOI:** 10.3390/biomedicines11072084

**Published:** 2023-07-24

**Authors:** Nima Nabavi, Seied Rabi Mahdavi, Mohammad Afshar Ardalan, Mohsen Chamanara, Reza Mosaed, Aline Lara, Diogo Bastos, Sara Harsini, Emran Askari, Pedro Isaacsson Velho, Hamed Bagheri

**Affiliations:** 1 Nuclear Medicine Research Center, Mashhad University of Medical Sciences, Mashhad 13944-91388, Iran; nima.nbv@gmail.com (N.N.);; 2 Radiation Sciences Research Center, AJA University of Medical Sciences, Tehran 14117-18541, Iran; 3 Department of Medical Physics, Radiation Biology Research Center, Iran University of Medical Sciences, Tehran 14117-18541, Iran; 4 Department of Internal Medicine, School of Medicine, AJA University of Medical Sciences, Tehran 14117-18541, Iran; 5 Department of Pharmacology, School of Medicine, AJA University of Medical Sciences, Tehran 14117-18541, Iran; 6 Department of Clinical Pharmacy, School of Medicine, AJA University of Medical Sciences, Tehran 14117-18541, Iran; 7 Hospital Sírio-Libanês, São Paulo 01308-050, Brazil; 8 Hospital do Câncer UOPECCAN, Cascavel 85806-300, Brazil; 9 Oncology Department, Hospital Sirio-Libanês, São Paulo 01308-050, Brazil; 10 BC Cancer Research Institute, Vancouver, BC V5Z 1L3, Canada; sharsini@bccrc.ca; 11 Sidney Kimmel Comprehensive Cancer Center, Johns Hopkins, Baltimore, MD 21231, USA; 12 Hospital Moinhos de Vento, Porto Alegre 90035-000, Brazil; 13 School of Medicine, AJA University of Medical Sciences, Tehran 14118-13389, Iran

**Keywords:** bipolar androgen therapy (BAT), prostate cancer, metastatic castration-resistant prostate cancer, supraphysiologic testosterone, prostate-specific membrane antigen, positron emission tomography (PET), novel hormonal agents (NHAs)

## Abstract

Androgen deprivation therapy (ADT) remains the cornerstone of advanced prostate cancer treatment. However, the progression towards castration-resistant prostate cancer is inevitable, as the cancer cells reactivate androgen receptor signaling and adapt to the castrate state through autoregulation of the androgen receptor. Additionally, the upfront use of novel hormonal agents such as enzalutamide and abiraterone acetate may result in long-term toxicities and may trigger the selection of AR-independent cells through “Darwinian” treatment-induced pressure. Therefore, it is crucial to develop new strategies to overcome these challenges. Bipolar androgen therapy (BAT) is one such approach that has been devised based on studies demonstrating the paradoxical inhibitory effects of supraphysiologic testosterone on prostate cancer growth, achieved through a variety of mechanisms acting in concert. BAT involves rapidly alternating testosterone levels between supraphysiological and near-castrate levels over a period of a month, achieved through monthly intramuscular injections of testosterone plus concurrent ADT. BAT is effective and well-tolerated, improving quality of life and potentially re-sensitizing patients to previous hormonal therapies after progression. By exploring the mechanisms and clinical evidence for BAT, this review seeks to shed light on its potential as a promising new approach to prostate cancer treatment.

## 1. Introduction

Prostate cancer (PC) continues to be a global public health concern. It is the most prevalent malignancy among males in 112 countries and the leading cause of cancer-related mortality in a quarter of the world’s nations, accounting for approximately 375,000 deaths every year [1,2]. Although the overall incidence of PC has remained stagnant in recent years, there has been a 2.5-fold increase in the diagnosis of metastatic disease, rising from 3.9% to 8.2%, between 2007 and 2018 [3].

Given PC’s dependence on androgens and the androgen receptor (AR) for growth, castration, either through surgery or medication, has been the backbone of treatment for metastatic disease. However, even when cancer initially responds to AR axis suppression, virtually all patients eventually progress to a state known as castration-resistant prostate cancer (CRPC). The Prostate Cancer Working Group 3 proposed the term “CRPC” for prostate cancers that progress clinically or radiographically despite castrate levels of serum testosterone (50 ng/dL) [4,5]. The reactivation of the androgen receptor mediates the transition toward CRPC through various adaptive mechanisms, enabling CRPC cells to survive even at castrate levels of circulating androgens. This was evidenced by studies showing that approximately one-fifth of specimens derived from CRPC patients still expressed the androgen receptor [6]. In an effort to further inhibit the AR pathway, novel hormonal agents (NHAs) were developed. The improved overall survival (OS) recorded upon utilizing NHAs, such as abiraterone (ABI) and enzalutamide (ENZ), in CRPC patients supports the notion that AR remains the primary driver of CRPC [7,8]. Despite their clinical benefit, however, resistance to these novel agents inevitably emerges, and further AR-targeted treatments often fail to achieve the desired response [9,10,11,12,13]. Therefore, there is an urgent and unmet need to develop new strategies to combat these AR-driven resistant cells. Furthermore, NHAs have recently received approval for the treatment of non-metastatic CRPC (nmCRPC) and metastatic hormone-sensitive PC (mHSPC), implying that these potent drugs will be deployed earlier in the course of the disease [14,15,16,17,18,19,20,21,22]. Although these drugs have been shown to delay the progression towards CRPC and there are studies that found no associations with more aggressive phenotypes, this paradigm shift may increase the evolutionary pressure through selecting AR-independent cells while increasing the likelihood of long-term toxicities, thereby emphasizing the further need for novel strategies to surmount these obstacles [23,24].

Bipolar androgen therapy (BAT) is one such approach that aims to exploit the overexpression of AR in CRPC cells [25]. This novel strategy involves the controlled manipulation of testosterone levels, alternating between supraphysiological and castrate states, achieved by continuing androgen deprivation therapy (ADT) in conjunction with periodic intramuscular testosterone injections. BAT was developed based on preclinical studies that demonstrated the inhibitory effect of supraphysiologic testosterone (SPT) on the growth of CRPC cells with AR overexpression [26]. This “paradoxical” effect of SPT in CRPC cells can be attributed to several possible underlying mechanisms, including the disruption of DNA relicensing, induction of double-stranded DNA breaks (DSBs), inhibition of wild-type AR expression, modulation of oncogene expression, and promotion of immune response [27]. The alternating design of BAT disrupts the adaptive autoregulation of AR in PC cells by depriving them of the sufficient time to respond to androgen levels in the environment. Cells expressing high levels of AR are susceptible to the effects of SPT, while cells with low basal AR levels or those that have downregulated their AR levels would not survive the subsequent castrate phase of the treatment cycle [28,29].

Several clinical trials have been conducted over the past decade to assess the efficacy and safety of BAT in metastatic prostate cancer [26,30,31,32,33,34,35]. Our objective is to provide a comprehensive review of the mechanism of action, underlying rationale, completed clinical trials, and upcoming investigations of BAT.

## 2. The Androgen Receptor (AR)

**Figure 1:**
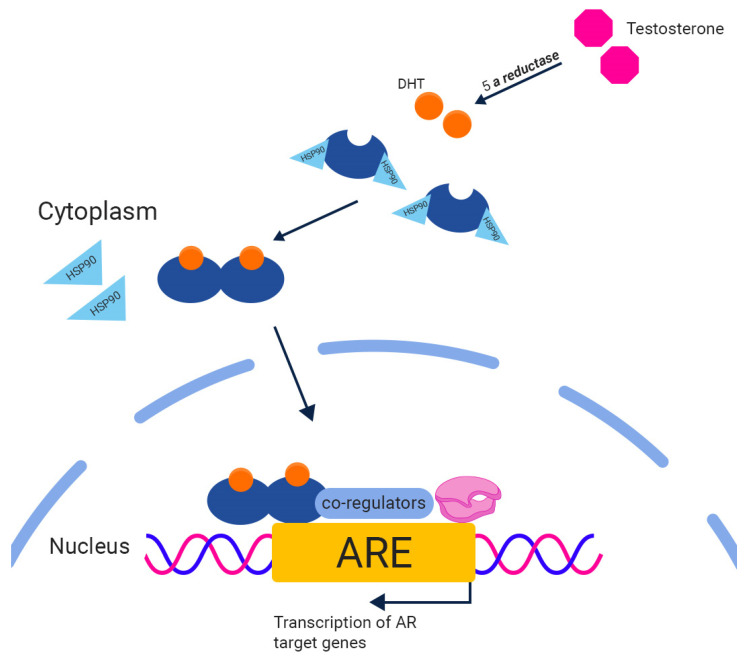
The androgen receptor signaling pathway. The 5-a reductase enzyme converts testosterone into the highly active dihydrotestosterone (DHT). Unbounded AR is located in the cytoplasm, chaperoned by a complex of proteins such as HSP90. Upon the binding of DHT, AR separates from chaperone proteins, dimerizes, and relocates to the nucleus, binding to androgen response elements (AREs) found in AR target genes.

AR is a nuclear steroid receptor that plays an essential role in the development of prostate tissue. This ligand-dependent transcription factor mediates the cellular effects of androgens, with testosterone and dihydrotestosterone being the primary agonists. Similar to other members of the steroid receptor superfamily, AR consists of four functional domains: An N-terminal domain (NTD), a DNA-binding domain (DBD), a C-terminal ligand-binding domain (LBD), and a flexible hinge that connects the DBD and LBD. In its unbound state, AR resides in the cytoplasm, chaperoned by a complex of heat shock proteins and immunophilins that protect it from degradation and induce a conformation with high ligand affinity. When a ligand binds to the AR, it dissociates from the chaperone complex, forms a dimer, and translocates to the nucleus. In the nucleus, the AR binds to androgen response elements (AREs) present in the promoter and enhancer regions of AR target genes [36,37,38] (Figure 1).

In normal prostatic epithelial cells, AR functions as a growth suppressor. Physiologically, the coupling of androgens to AR causes prostate epithelial cells to enter G0 cell arrest and promotes their differentiation into quiescent luminal cells. In rodent models, the downregulation or deletion of AR has resulted in hyperplastic growth and poor differentiation of the epithelial compartment. This suppressive effect is partly mediated by the transcriptional downregulation of c-Myc. Cells that constitutively transcribe *c-Myc* evade AR-induced growth suppression, indicating that c-Myc downregulation is required in this process. Additionally, the knockdown of *c-Myc* inhibits the growth of prostatic epithelial cells, establishing a causal relationship between its transcription and AR-mediated proliferation block [39,40].

**Figure 2:**
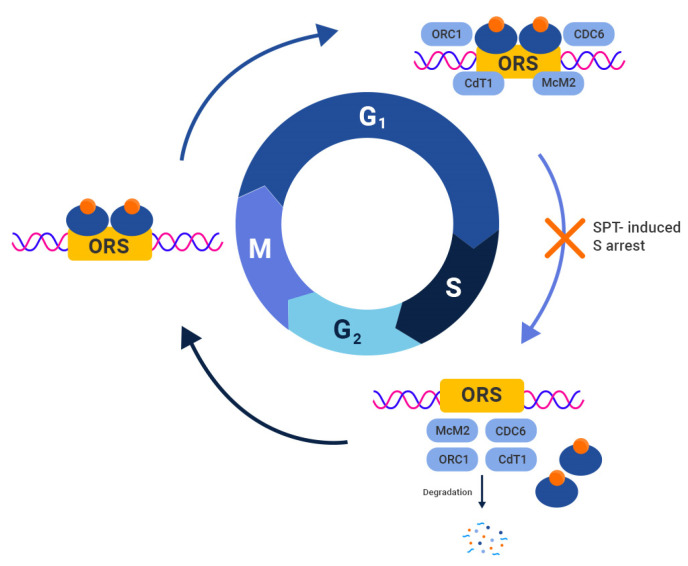
Oncogenic role of AR as a licensing factor and SPT-mediated disruption of DNA licensing. AR acts as a licensing factor for DNA replication in prostate cancer. During early G1, bounded AR joins the origin of replication sites (ORS), which are bound by the origin recognition complex (ORC). Then, cell division cycle 6 binds the ORC, which is necessary for loading MCM proteins and CDT1, forming the pre-replication complexes required for the G1-dependent DNA licensing. After the genome is replicated in the S-phase, replication complexes are removed from ORS during mitosis and degraded to ensure that ORS is available for subsequent relicensing. It has been hypothesized that upon SPT administration, ligand-dependent stabilization of AR during mitosis might prevent its degradation in the M phase, which disrupts the relicensing process, resulting in subsequent G1/S arrest.

On the other hand, AR functions as an oncogene in prostate cancer cells, promoting cell growth and survival. While the exact mechanism behind this oncogenic conversion is not fully understood, it appears that AR acquires an oncogenic gain of function in regulating *c-Myc* and DNA licensing, both of which are required for malignant cell proliferation. In PC-derived cell lines, the inhibition of AR resulted in growth arrest accompanied by undetectable *c-Myc* expression. These cells resumed the cell cycle when 1 nM R1881, a potent synthetic androgen, was introduced to the medium. This re-entry was accompanied by a rapid increase in c-Myc protein expression, indicating that AR signaling upregulates *c-Myc* expression in PC [41]. During the carcinogenesis of PC, AR assumes the role of a licensing factor for DNA replication. This hypothesis arose from the observation that AR forms complexes with known licensing factors at DNA replication origins in PC cells [42,43]. Licensing factors need to be degraded in each cell cycle to allow for their relicensing in the subsequent cycle (Figure 2).

Litvinov et al. documented that AR levels fluctuate according to cell-cycle phases and AR degrades during mitosis in PC cell lines—a characteristic not observed in normal human stromal cells where AR solely acts as a transcriptional factor [44]. Additionally, AR-signaling aberrations involve DNA alterations as well. One such example is the fusion of the androgen-responsive promoter of *TMPRSS2* to ETS transcription family members (*ERG* or *ETV1*), which results in the overexpression of these transcription factors in PC. However, the incidence of such fusions varies significantly based on race and ethnicity, highlighting the heterogeneity of genomic processes in PC [45,46,47,48,49].

Through various AR-related mechanisms, prostate cancer eventually escapes ADT and progresses toward castration resistance [50,51,52,53,54,55,56,57,58,59,60,61,62,63,64]. Among these adaptive strategies are AR overexpression and gene amplification. AR expression has been found to be 27- to 90-fold higher in CRPC cell lines compared to normal prostate epithelial cells and up to 30-fold higher than in localized PC patients who have not received androgen-targeted therapies. These mechanisms, which have been shown to be acquired molecular changes rather than inherent genetic aberrations, enable CRPC cells to inversely regulate their AR levels in response to circulating androgen levels [28,61,62]. AR splice variants (ARVs) are also involved in the hormonal resistance of PC [64]. ARV7, the most abundantly expressed ARV in human cell lines and clinical samples, has been found to be significantly higher in patients who do not respond to ENZ [63]. Its expression has been associated with lower PSA response rates and a shorter median OS in patients treated with ADT and NHAs [65].

The observation that PC continues to rely on AR signaling even in a castrate environment led to the development of NHAs that inhibit AR more potently, such as abiraterone, enzalutamide, apalutamide, and darulotamide. The fact that NHAs produce clinical benefits and prolong OS in CRPC patients confirms that AR-signaling remains the primary driver of PC after progression to castration resistance. Despite the initial response, the majority of patients eventually develop resistance to these agents as well. When alternative NHAs were administered as a third-line therapy to patients who had progressed on other NHAs, only a modest and brief response was observed [9,10,11,12,13]. Thus, there is a need for novel AR-targeted agents to effectively curb PC progression. The recent approval to use NHAs to treat nmCRPC and mHSPC may trigger the transition towards AR-resistant cells and cause long-term toxicities, highlighting the need for new approaches to overcome such challenges.

## 3. Preclinical Studies Utilizing SPT

Through his seminal studies, Dr. Huggins was the first to exploit the interplay of androgens and prostate cells therapeutically, demonstrating the beneficial impact of castration in prostate cancer. By utilizing serum phosphatases as biomarkers, he discovered the dependence of PC on androgens for proliferation and dissemination. While depriving cancerous cells of androgen halted disease progression, exogenous testosterone seemed to serve as “fuel on the fire” [66]. This research revolutionized how prostate cancer was viewed and treated, paving the way for future prostate cancer treatments targeting androgen-driven pathways [67,68].

Dr. Huggins was also the first to reveal that excessive hormones can “conspicuously” induce tumor regression. He demonstrated that progesterone, a promoter of mammary cancer growth, when administered in tandem with estradiol in supraphysiological doses, causes a significant decrease in the number of cancers, even “extinguishing” it in almost 30% of rats [69]. He attributed this paradox to the interference with the hormonal mechanisms of cancer, introducing “hormone interference” as a novel approach in the fight against cancer [69]. On this account, the effect of SPT on PC has been investigated. There are relatively few established human PC cell lines, among which the most notable are DU145, PC3, and LNCaP cells [70]. The majority of in vitro studies have used LNCaP or one of its subclones. This androgen-sensitive cell line, derived from a lymph node metastasis of PC, is unique in that it expresses a mutant AR and thus can model different stages of PC progression [71]. LNCaP cell lines exhibit a biphasic response to the addition of androgens to the medium; while low doses of R1881 (0.1 nM) doubled cell growth, supraphysiological doses (1–100 nM) inhibited proliferation [72,73,74]. To induce androgen sensitivity in AR-negative PC3 cells, full-length *AR* DNA was transfected into these cells. Androgens inhibited the growth of these AR-transfected PC3 cell lines as well [75,76].

In vivo studies have also shed light on the potential benefits of exogenous androgens in the treatment of resistant prostate cancerous cells. Through the serial passage of androgen-sensitive LNCaP 104-S cells in a castrate environment 100 times, LNCAP 104-R cells emerged. These cells express AR but are not androgen-dependent for proliferation. The injection of LNCAP 104-R cells into castrated nude athymic mice resulted in the development of tumors. The growth of LNCaP 104R-derived tumors was prevented by implanting testosterone propionate pellets and induced regression in established tumors of this line—an effect not seen in tumors derived from LNCaP 104-S cells or the AR-negative PC-3 lines [77]. Remarkably, the removal of testosterone implants or initiation of finasteride, a 5-a-reductase inhibitor, led to the regrowth of regressed tumors. Although LNCaP 104 R tumors initially regressed in response to SPT, they eventually adapted and evolved into R1Ad cells, which demonstrated androgen-stimulated growth. Surprisingly, castration levels of androgen inhibited the growth of these androgen-dependent R1Ad cells, indicating that SPT is capable of reverting resistant cells to an androgen-sensitive state [78].

**Figure 3:**
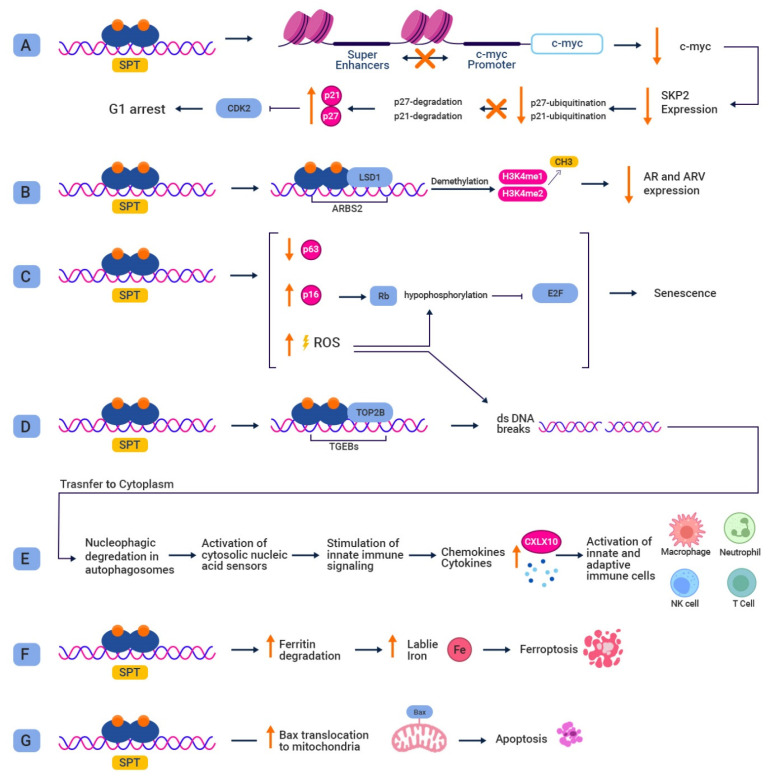
Proposed mechanisms for growth-inhibitory effects of SPT. (**A**) SPT is hypothesized to disrupt the interactions between distal super enhancers and the c-Myc promoter, which results in c-Myc downregulation and its target gene S-phase kinase-associated protein 2 (SKP2). SKP2 suppression entails the loss of the ubiquitin-mediated degradation of cyclin-dependent kinase (CDK) inhibitors p27 and p21, causing G1 arrest. (**B**) By activating AR signaling, SPT may cause transcriptional repression at specific binding sites in the second intron of the *AR* gene (ARBS2) through recruiting lysin-specific histone demethylase 1 (LSD1) and the subsequent demethylation of activating histone marks such as H3K4 me1-2, which results in a reduced expression of full-length AR and its spliced variants. (**C**) SPT-mediated AR activation may induce senescence through multiple mechanisms: decreasing the expression of p63, a protein which opposes senescence; promoting the production of ROS, which leads to Rb hypophosphorylation and the subsequent suppression of E2F target genes; increasing the expression of p16, which promotes the formation of senescence-associated heterochromatic foci (SAHF) through the p16-Rb-E2F axis. (**D**) SPT may induce ds-DNA breaks by the recruitment of topoisomerase II beta (TOP2B) to AR target genes. Increased ROS production potentially plays a role in conversion of transient ds-DNA breaks into true ones. (**E**) Autophagosome-mediated nucleophagy of damaged DNA appears to activate cytosolic nucleic acid sensors, stimulating the innate immune system, which results in cytokine and chemokine release including CXCL10 and the eventual activation of immune cells. (**F**) Through increasing ferritin degradation, SPT has been suggested to increase the labile iron pool, leading to lipid peroxidase-mediated cell death or ferroptosis. (**G**) SPT administration seems to facilitate Bax protein’s translocation to mitochondria, thereby promoting apoptosis.

Although the exact mechanism behind the paradox of SPT is not fully understood, several contributory mechanisms have been proposed (Figure 3). Genetic screens and the transcriptional profiling of PC cells have not revealed a single dominant pathway responsible for mediating the growth-suppressive effects of SPT, suggesting that multiple concurrent mechanisms are involved [79]. Early in vitro investigations revealed that SPT-induced growth inhibition is associated with reduced c-Myc expression [73]. Mechanistically, Guo et al. demonstrated that SPT represses *c-Myc* by disrupting the interactions between distal super-enhancers, such as *PCAT1* and *PVT1*, and the *c-Myc* promoter. This repression coincided with conformational changes in the 8q24 topologically-associated domain [80]. A recent study validated the role of c-Myc downregulation in growth inhibition by SPT, but only in cells expressing high levels of AR. Knockdown of AR in SPT-sensitive LNCaP cells prevented c-Myc downregulation, whereas AR overexpression in SPT-resistant LAPC4 and 22Rv1 lines resulted in SPT-induced c-Myc downregulation. This indicates that elevated AR expression prior to SPT is both necessary and sufficient to cause c-Myc downregulation. Furthermore, forced continuous c-Myc expression only partially rescued cells from this growth-inhibitory impact, confirming that decreased c-Myc expression comprises just one of the numerous mechanisms by which SPT inhibits growth [81]. The downregulation of c-Myc results in the suppression of its target genes, one of which is SKP2, the substrate-recruiting subunit of the SCF E3 ligase complex. SKP2 plays a role in phosphorylating cyclin-dependent kinase inhibitors p27 and p21, marking them for ubiquitination and subsequent degradation [82]. By suppressing SKP2, the ubiquitin-mediated degradation of CDK inhibitors is hindered, leading to the inhibition of cyclin E/CDK2 complex activity and inducing G1 arrest [83,84,85,86].

Given the significant role of adaptive AR overexpression and AR splice variants in the progression toward CRPC, reversing these phenomena may be another mechanism by which SPT induces growth inhibition. AR mRNA and protein levels in LNCaP and VCaP cells have been shown to decrease in response to androgens in vitro and in vivo [73,87,88,89,90]. While investigating the underlying mechanisms of such suppression, Cai et al. identified a highly conserved binding site in the second intron of the *AR* gene (ARBS2), which regulates *AR* expression according to the abundance of androgens in the environment. By recruiting LSD1 to ARBS2 and causing the subsequent demethylation of H3K4 me1-2, ligand-bound AR reduced the expression of the *AR* gene. LSD1 also mediated the AR-induced downregulation of AKR1C3 and HSD17B6, providing additional evidence that ligand-bound AR controls AR activity directly through negative feedback loops [91]. Repeated passages of JDCaP xenografts (derived from CRPC skin metastases) in a castrate environment resulted in the emergence of JDCaP-hr cells that overexpressed AR and AR-V7. Administration of exogenous testosterone to JDCaP-hr cells drastically reduced AR and AR-V7 mRNA and protein levels in a dose-dependent manner, both in vitro and in vivo [92]. This decrease in AR splice variants, however, has yet to exhibit a therapeutic benefit in clinical settings.

Another proposed mechanism by which SPT may inhibit cell growth is through the disruption of DNA licensing and replication processes. As previously stated, AR gains function as a licensing factor for PC’s DNA replication. During early G1, ligand-bound AR joins the origin of replication sites (ORS), which are first bound by the highly preserved origin recognition complex (ORC), consisting of six subunits (ORC1-6). Following that, cell division cycle 6 (CDC6) binds to ORC, which is needed to load a heteroheptameric ring composed of MCM 2–7 proteins and the replication factor CDT1, completing the formation of pre-replication complexes required for G1-dependent DNA licensing. Following DNA licensing, the cell enters the S-phase, during which its genome is replicated once. This “one replication per cycle policy” is enforced by cyclin-dependent kinase (CDK)-induced inactivation, nuclear export, and the eventual proteasomal degradation of licensing factors. The removal of replication complexes from ORS during mitosis ensures that ORS is available for relicensing in the subsequent G1 phase. However, under SPT conditions, it has been proposed that ligand-bound AR is stabilized during mitosis, preventing degradation. Consequently, AR remains bound to ORS, rendering it inaccessible for relicensing and leading to G1/S arrest [93] (Figure 2).

Another potential mechanism by which SPT inhibits proliferation is through the induction of DNA damage. Although the precise underlying mechanism of such an impact is obscure, studies suggest that ligand-bound AR recruits topoisomerase II beta (TOP2B), resulting in TOP2B-mediated double-strand DNA breaks (dsDBs) at AR target genes [94]. These dsDBs are recognized by DNA repair proteins such as Ku70, Ku80, PARP1, ATM, and DNA-PK, which are recruited to these sites [95]. According to Chatterjee et al., SPT exposure induces dsDBs and significantly downregulates genes that encode DNA damage repair proteins, resulting in cell-cycle arrest, senescence, and apoptosis. In their study, the extent of SPT-induced DNA damage was related to AR overexpression and ligand concentrations. BRCA2-deficient cell lines and patient-derived xenografts were more susceptible to the damaging effects of SPT, implying that patients harboring DNA repair mutations may benefit from this accentuated efficacy [96]. Additionally, increased production of reactive oxygen species may convert the initially transient DNA breaks caused by AR transcription into true double-strand breaks [97]. Furthermore, the illegitimate repair of such unresolved dsDBs can result in structural genomic rearrangements such as *TMPRSS2:ERG* fusions [94,95].

SPT administration has been found to promote apoptosis as well. In experiments using castration-resistant MOP cell lines derived from LNCaP cells, treatment with 100 nM R1881, a potent synthetic androgen, significantly reduced the mitotic index and induced apoptosis in nearly 40% of the cells, compared to less than 0.4% in control cells [98]. Lin et al. demonstrated that this proapoptotic effect is mediated by facilitating the translocation of the Bax protein to the mitochondria. AR knockdown terminated Bax-mediated apoptosis in AR-positive cells, while the transfection of *AR* into AR-negative PC-3 cells made them vulnerable to exogenous Bax, demonstrating that AR is essential for this Bax-induced apoptosis [99]. Moreover, AR sensitized castration-resistant LNCaP cell lines to genotoxic stress through the PIRH2-p53-p21 axis [100].

Irreversible cell-cycle arrest (senescence), autophagy and autophagosome-mediated immune cell activation have also been proposed as contributors to the inhibitory effect of supraphysiological androgens. Senescence, which appears to be inhibited in cancerous cells, acts as a preventive mechanism against malignant proliferation in premalignant cells [101]. Therefore, reactivating this process may induce tumor suppression and has been proposed as a potential therapeutic target [102]. SPT-induced senescence in PC cell lines has shown to be dose-dependent, as measured by senescence-associated beta-galactosidase activity. This AR-induced senescence appears to be mediated via multiple underlying mechanisms acting in concert. AR signaling promotes ROS production, which causes Rb hypophosphorylation and, as a result, the suppression of E2F target genes. Another mechanism by which SPT induces irreversible cell arrest is by reducing p63 expression, a protein from the p53 family that opposes senescence [103]. By examining the formation of senescence-associated heterochromatic foci (SAHF), Roediger et al. confirmed androgen-induced senescence and identified the p16-Rb-E2F axis as a parallel contributing mechanism. Furthermore, SPT-induced cell-cycle arrest was observed as early as 3 h post-administration. In light of this, they hypothesized that a non-genomic rapid signaling response is involved and identified Src tyrosine kinase and the downstream pathways Akt-PI3K and Akt-mTOR as mediators [104]. Intriguingly, SPT upregulated autophagy activity in PC cell lines, as evidenced by the conversion of LC3-1 to LC3-2, a key marker of autophagy [104]. This SPT-induced autophagy was confirmed in a recent study that found an increase in the number of autophagosomes and autophagy influx. Ferritinophagy, or ferritin molecule degradation, has been proposed as a mediating mechanism. The degradation of ferritin increases the labile iron pool, leading to lipid peroxidase-mediated cell death, termed as ferroptosis. SPT reduces ferritin in a dose-dependent manner by co-recruiting it with LC3 B-positive autophagosomes. Another parallel autophagy-mediated mechanism by which SPT suppresses cell growth appears to be nucleophagy, where damaged DNA is shuttled to autophagosomes for nucleophagic degradation, a feature previously observed in chemo- and radiotherapy [105,106,107]. The presence of DNA in the cytoplasm activates cytosolic nucleic acid sensors, triggering innate immune signaling and resulting in the secretion of cytokines and chemokines such as CXCL10, which leads to the activation of innate and adaptive immune cells [105].

## 4. Bipolar Androgen Therapy (BAT)

Following the discovery of the beneficial effects of castration in prostate cancer, subsequent studies administered exogenous testosterone primarily to confirm that these favorable results were indeed mediated by the decreased testosterone levels [67]. While many studies reported that testosterone administration reversed the benefits of castration and resulted in disease progression, others observed improvements [108,109,110,111,112,113,114]. Notably, two case reports documented significant improvements in two mCRPC patients following testosterone treatment [115]. Although these early reports showed promise, a thorough investigation of testosterone as a therapy for prostate cancer was not conducted until the last two decades. Multiple studies demonstrated the safe use of testosterone replacement therapy to treat hypogonadism in prostate cancer patients, without exacerbating the disease [116,117,118,119,120]. Building on these findings, two phase 1 clinical trials examined the efficacy of transdermal testosterone in patients with CRPC. Although the results of both trials were subpar, with only one patient achieving a decline in PSA of 50% or more between the two studies, it was noteworthy that testosterone administration was well-tolerated and did not lead to disease progression or the worsening of symptoms. This suggests that testosterone can be safely administered in patients with castration-resistant prostate cancer. However, it is important to mention that neither of these studies were able to achieve supraphysiological levels of testosterone [121,122].

Samuel Denmeade and John Isaacs saw the adaptive autoregulation of AR in CRPC and its oncogenic gain of function in the DNA relicensing process as potential liabilities that could be therapeutically exploited [25,28]. Based on preliminary research uncovering the paradoxical growth-inhibitory effects of SPT, they proposed BAT as a novel strategy to treat CRPC, which involves acute cyclical alternations between supraphysiologic and castrate levels of androgen [26]. This is achieved by administering 400 milligrams of intramuscular testosterone cypionate every 28 weeks while patients continue to receive ADT concurrently. The proposed rationale behind BAT is that SPT would inhibit the proliferation of cells that had progressed on ADT through AR overexpression, partly by disrupting the relicensing process. Meanwhile, cells with low AR expression, either basally or through adaptive downregulation, would be susceptible to the subsequent castrate phase of the treatment cycle [29].

## 5. BAT Studies

BAT was initially tested in castrated NOG mice using xenografts of LNCaP/A-cells, which were adapted to grow in androgen-ablated environments and exhibited an overexpression of AR 75-fold higher than normal epithelial prostate cells. Animals were implanted with testosterone capsules, which rapidly increased testosterone levels from castration to supraphysiologic levels. After two weeks, the testosterone capsules were removed, causing a drop in circulating androgens to ablated levels. Subsequently, the testosterone capsules were re-implanted after the mice had been in a castrate environment for two weeks. BAT inhibited growth by more than 70%, primarily due to increased cell death rather than decreased proliferation. These successful in vivo and in vitro studies paved the way for the first clinical study of BAT [26].

The first clinical study that utilized BAT was conducted to evaluate its safety and efficacy on asymptomatic CRPC patients who progressed on long-term ADT [26] (Table 1). This pilot single-arm study combined BAT with etoposide, a TOP2B inhibitor. This combination was chosen based on studies that suggested SPT induces TOP2B-mediated dsDNA breaks; thus, combining it with an agent that traps catalytically active TOP2B on the DNA would hypothetically result in synergism. To do so, patients received three 28-day cycles of BAT plus oral etoposide at a dose of 100 mg/day for the first 14 days, in combination with intramuscular testosterone cypionate at an FDA-approved dose of 400 mg on the first day of each cycle. Throughout the study, the patients remained on ADT, allowing their androgen levels to oscillate between supraphysiological and castrate or near-castrate levels. Patients whose PSA declined after three cycles were eligible to enter the second phase of the study, in which BAT monotherapy was administered until progression. PSA levels decreased in half of the patients (7/14), with four experiencing a 50% decline (PSA50). For these seven responders, the median duration of clinical benefit was 343 days. Furthermore, an objective response was observed in half of the patients with a RECIST-evaluable lesion (5/10). Three distinctive patterns were seen across the patients regarding PSA: (A) an overall increase (7/14); (B) an initial spike after the first dose followed by a decline (3/14); (C) a steady decline (2/14).

Patients were re-challenged with androgen-ablative therapies after progressing on BAT; surprisingly, all patients responded to some extent, implying that BAT may have the potential to reverse androgen resistance and re-sensitize patients to such agents [26]. Out of the 16 initially enrolled patients, two were withdrawn from the study and did not complete the first phase, one due to grade 2 priapism and the other due to pneumonia-related death. The remaining 14 patients did not experience new pain or urinary obstruction due to PC, and the treatment regimen was well-tolerated. The majority of the adverse events occurred during the first phase of the study and were known etoposide side effects such as nausea, fatigue, alopecia, edema, and neutropenia. Adverse events (AEs) were rare and low-grade during BAT monotherapy, with only four subjects experiencing mostly grade 1 events [26].

Following the encouraging results of this pilot study, a phase 2 single-arm study was conducted to assess the efficacy and safety of BAT as a first-line therapy in men with asymptomatic androgen ablation naive prostate cancer (BATMAN study) [30]. BATMAN’s design included a six-month “lead-in” ADT monotherapy. Patients who had a PSA of 4.0 ng/mL or achieved a 50% or higher decrease in PSA (PSA50) at the end of this phase were eligible to receive two rounds of “BAT-ADT”, consisting of three cycles of BAT plus ADT followed by 12 weeks of ADT monotherapy. After the 18-month study period, patients continued to receive ADT therapy. This design was chosen to determine whether BAT can prolong the castration-sensitive state of PC cells by interfering with AR’s adaptive autoregulation. Out of the 33 patients enrolled, 29 were eligible to enter the BAT-ADT phase, with 3 dropping out due to progression. Nearly 60% of the subjects (17/29 patients) achieved a PSA ≤ 4 ng/mL. Eight out of ten patients with a RECIST-evaluable disease showed a clinical response [30].

Recently, the results of the BATMAN study were updated and published after a median follow-up of nearly eight years [31]. The median overall survival (OS) for the responder group (those with a PSA 4 at the end of the 18-month study) has not yet been reached, compared to 43 months for non-responders (*p* = 0.002). After this monitoring period, no serious safety concerns or long-term adverse events were reported. A quarter of the patients were still hormone sensitive. After progressing on BAT, 19 patients received second-line therapies (8/19 ABI, 11/19 ENZ). PSA50 was achieved in 94.4% of these patients, which compares favorably to 78% for ENZ in the PREVAIL study and 62% for ABI in the COU-AA-302 study. The time to progression on ADT in the BATMAN study was 20.6 months, nearly double that of the two aforementioned studies [31]. Although such encouraging results may reflect a selection bias toward recruiting more androgen ablation-sensitive patients, they may imply that BAT prolongs the PC’s sensitive window to second-line therapies, which is consistent with the findings of the TRANSFORMER study [33]. Intriguingly, patients with peak PSA values of <9 ng/mL after three months of BAT therapy had a significantly longer PFS than those with values ≥ 9 (NR vs. 20.6 months, HR: 0.122, *p* < 0.001) [31].

In the phase 2 clinical trial TRANSFORMER, 195 asymptomatic metastatic CRPC patients progressing on ABI were randomly assigned in a 1:1 ratio to receive either BAT or ENZ until clinical or radiological progression. Upon progression, asymptomatic patients who still met the eligibility criteria had the option to crossover to the alternate treatment. The crossover study assessed the time to PSA progression and the time from randomization to progression after crossing over (PFS2). Despite the fact that TRANSFORMER was not sufficiently powered to be a non-inferiority study, BAT and ENZ were similar in terms of PFS (5.7 months each), time to PSA progression (2.8 vs. 3.8 months), and PSA50 response (28.25% vs. 25.5%). Intriguingly, BAT was more effective in patients with a shorter duration of response to ABI (<6 months), implying that BAT may play a role in reversing the lineage plasticity in PC cells with diminished AR signaling. Approximately 40% of the subjects switched to the opposite treatment after progression. Patients who crossed over to ENZ after receiving BAT (BAT→ENZ) had better outcomes than those who crossed over to BAT after receiving ENZ (ENZ→BAT), with higher PSA50 response rates (77.8% vs. 21.3%), objective response rates (28.3% vs. 7.3%), and longer PFS2 (28.2 vs. 19.6 months), all favoring the BAT→ENZ sequence. The median time to progression for BAT→ENZ was 10.9 months, more than double that of ENZ (3.8 months) when used immediately after progression on ABI. Furthermore, compared to ENZ alone, BAT→ENZ showed higher PSA50 response rates (78% vs. 25%) and objective response rates (29.4% vs. 4%). These findings lend support to the hypothesis that BAT can revert resistant PC cells into a castration-sensitive state, significantly improving the extent and duration of response to ENZ [33].

The RESTORE trial was a multicohort, phase 2 clinical trial that investigated the effects of BAT in patients with asymptomatic metastatic CRPC who had progressed on ENZ (Cohort A), ABI (Cohort B), or first-line castration-only therapy (Cohort C). Each cohort recruited 30 patients, for a total of 90 patients.

In Cohort A, 30% of subjects achieved a PSA50 response after a median of six BAT cycles. Among the 21 patients who were re-challenged with ENZ, 15 demonstrated a PSA50 response. BAT resulted in a complete or partial radiographic response in 50% of patients with RECIST-evaluable disease, and the median clinical or radiographic PFS was just under nine months. The median PFS2 in this cohort was 12.8 months. These results further accentuate the re-sensitizing benefits of BAT as an intervening therapy following the progression of patients on ENZ [34].

In the post-ABI cohort, 17% (5/29) of the subjects attained PSA50 in response to BAT, while only 16% (3/19) achieved PSA50 upon ABI re-challenge. The objective response rate was 29%, and the clinical/radiographic PFS on BAT was 4.3 months. Similar to the PSA50, the crPFS and the PFS2 after the ABI re-challenge were significantly shorter compared to the post-ENZ cohort. It is important to note that the RESTORE trial was not designed to directly compare the cohorts, and therefore, these differences could be attributed to a lack of statistical power. Despite the potential limitations of the study design, these findings suggest that BAT may not be as effective as a re-sensitizer for ABI compared to ENZ [34].

In the RESTORE Cohort C, 14% (4/29) of the patients achieved PSA50. The objective response rate in this cohort was 31%, which is comparable to the reported 36% for ABI in the COU-AA-302 trial but inferior to ENZ’s 59% in the PREVAIL trial. Moreover, the radiographic progression-free survival (rPFS) with BAT in this cohort was 8.5 months, shorter than the approximately 11 months reported for the other two drugs. These findings suggest that when used as a first-line therapy, BAT may be less effective than NHAs. Nonetheless, the post-BAT response to subsequent NHAs was substantial, with 94% and 83% of patients achieving PSA50 and PSA90, respectively. In fact, the median PSA-PFS has yet to be reached after a median follow-up of 11.2 months, and 78% of patients have yet to progress at the 18-month mark. Such results supersede those of the PREVAIL and COU-AA-302 trials, implying that it may be best to avoid considering BAT as a first-line stand-alone in CRPC treatment. Instead, PSA-PFS2 may serve as a more accurate indicator of BAT’s clinical benefits, as evidenced in this study, where the median PSA-PFS2 has not yet been reached after a median follow-up of 26.2 months. This study found no correlation between the length of BAT therapy and the subsequent response to NHAs, suggesting that even brief exposure to BAT enhances subsequent response to these agents [35].

Two BAT combination trials were recently completed. The first of these studies is the combination of BAT with the PARP1 inhibitor Olaparib [123]. The rationale behind this combination is based on preclinical studies demonstrating that PARP inhibition augments SPT’s dsDNA-damaging effects while inducing senescence. The trial enrolled 36 asymptomatic mCRPC patients who had progressed on ABI and/or ENZ were enrolled; half of the enrolled patients were required to have at least one pathogenic alteration in an HRR gene. Among the patients, 44% achieved a PSA50 response (16/36), and the radiographic response rate was 54.5% (7/13). Notably, there was no significant difference in PSA50 or radiographic response between patients with intact HRR genes and those with HRR gene alterations. The median PSA PFS was reported to be 7 months, median crPFS was 13 months, and median OS was 26 months. These outcomes are better than those of BAT monotherapies, suggesting that this combination may result in more favorable clinical outcomes. However, larger studies are needed to confirm these findings. Even though 13% of the subjects experienced serious adverse events, including one stroke and one myocardial infection, the treatment was well-tolerated, and patients experienced improved QoL status, especially regarding erectile function score [123].

The second of these recently completed clinical trials is the COMBAT-CRPC study, which investigated the combination of BAT with nivolumab [124]. Nivolumab is a fully human monoclonal IgG4 PD-1 antibody that enhances T-cell pro-inflammatory function and antitumor immune responses by inhibiting the binding of PD-L1 to PD-1. This combination was selected based on the observation of a dramatic response to immune checkpoint blockade therapy in patients who had previously received BAT therapy. In fact, a retrospective review at Johns Hopkins Hospital found 41 mCRPC patients receiving immune checkpoint blockade and stratified these patients based on whether or not they had previously received BAT therapy. Intriguingly, two-thirds of patients who had previously received BAT therapy achieved PSA50 (4/6), compared to only 11.4% of those who had not (*p* = 0.008). On this basis, it was hypothesized that BAT might have a “priming” effect on the immune system in addition to promoting DNA damage, thereby enhancing the clinical response to immune checkpoint blockade synergistically. PSA50 was achieved in 40% of the patients (18/45), ORR was 23.8%, and median rPFS was 5.7 months. Most AEs were of low grade (<grade 2), with edema, nausea, and back pain being the most commonly reported. These preliminary results were published as an abstract, and the molecular biomarker and response predictor analysis is yet to be published [124].

## 6. Ongoing Clinical Studies

Given the variety of mechanisms by which SPT inhibits malignant proliferation, multiple ongoing studies are utilizing different combinations and sequences with the hope of augmenting and prolonging the efficacy of BAT (Table 2). The phase 2 clinical trial HiTech aims to evaluate the efficacy of BAT plus Carboplatin in mCRPC patients (NCT035522064). Carboplatin is a platinum-based chemotherapy drug that interferes with DNA replication through inter-strand cross-linking, resulting in G2/M phase cell accumulation and triggering apoptosis. The study is recruiting mCRPC patients with confirmed homologous recombination defects in germline and/or somatic DNA analysis.

There are currently two ongoing studies investigating the sequential use of BAT with a NHA. The first among these studies is ExBat (ExTreme Bipolar Therapy), which is a multi-center, single-arm phase 2 clinical trial, testing the sequential combination of BAT and darolutamide in mCRPC patients progressing on ABI(NCT04558866). Enrolled patients are set to undergo six 63-day cycles until progression and/or unacceptable toxicity. Each patient is subject to receive 400 mg of intramuscular testosterone cypionate on day 1 of each cycle, sequenced by 1200 mg of oral darolutamide (two 300 mg tablets q12hrs) from day 29 to 56, followed by a washout period of 7 days. Another study exploring the alternating use of BAT and an NHA is called STEP-UP (NCT04363164). mCRPC patients who had progressed on ADT+ABI or ABI after ADT progression will be randomized 1:2:2 and stratified based on their previous treatment status and the duration of response to ABI. In arm A, patients will receive ENZ continuously, whereas, in arms B and C, patients will undergo sequential treatment with BAT and ENZ in a fixed and variable manner, respectively. These sequencing studies are based on clinical observations regarding BAT’s capacity to re-sensitize CRPC to AR-targeted therapies, as discussed previously. BAT has been shown to revert AR overexpression and the expression of ARVs, one of the mechanisms by which PC escapes and resists AR inhibition. This was confirmed in the COMBAT trial, as all the collected samples exhibited AR downregulation after BAT therapy.

The BAT-RAD study is a single-arm phase 2 clinical trial that is testing the combination of BAT with radium-223, an alpha-emitting radiopharmaceutical that targets bones and has been shown to foster survival benefits in CRPC patients with bone metastasis only (NCT04704505). The rationale behind this combination is that both radium-223 and BAT cause double-stranded DNA breaks, which may improve the efficacy of BAT. This study is expected to be completed by 2027.

By the time this review is being written, there are no ongoing studies evaluating the combination therapy of BAT with taxanes. Conducting studies that utilize both these therapies might augment BAT’s efficacy or uncover other possible parallel mechanisms by which BAT works.

## 7. Safety

Concerns regarding the safety of bipolar androgen therapy (BAT) were initially raised due to the dependence of prostate cancer cells on androgens and the potential for exacerbating symptoms and stimulating tumor growth. These concerns were based on studies that suggested testosterone administration can particularly exacerbate the pain of bone metastases. On this account, patients suffering from pain requiring opioids and those with tumors in sites that could jeopardize patient safety in case of tumor flare (such as impending spinal cord or urethral compression and bone fractures) were excluded from BAT trials. With this cautious approach, BAT has been shown to be relatively safe and well-tolerated in asymptomatic metastatic castration-resistant prostate cancer (mCRPC) patients. Adopting such a precautionary approach, BAT has been shown to be relatively safe and remarkably well-tolerated in asymptomatic mCRPC patients. 

A recent systematic review evaluated the safety and tolerability of BAT across five trials encompassing more than 200 patients. This study reported BAT-induced AEs to be relatively rare, and when they did occur, they were primarily mild to moderate. Fatigue was the most common low-grade (1–2) AE, occurring in 13% of the patients. Other low-grade AEs with an incidence of 5% or above were: musculoskeletal pain (10.89%), edema (9.41%), nausea (8.42%), breast tenderness (7.43%), and increased hemoglobin levels (5.45%). The most common severe (grade 3–4) BAT-related AEs included hypertension (1.49%), pulmonary embolism (1.49%), and lower back pain (0.99%) [125].

Given that testosterone retains fluids in the body, antihypertensive drugs may need readjustment while on BAT, and patients with congestive heart disease should be treated with extra caution. Moreover, cardiovascular events are among the listed AEs of testosterone cypionate. Few heart attack and stroke incidents have been noted in BAT trials, though it was unclear whether BAT was the cause. Nevertheless, given the importance of this matter, patients with a known history of underlying cardiac disease should be monitored more closely. Another point to take into account is the method by which androgen deprivation therapy is achieved. Although both LHRH agonists and GnRH antagonists suppress testosterone levels effectively, they do so through different mechanisms. This difference in mechanism of action may translate into different side effect profiles. In line with this, in a pooled analysis of 2633 patients, GnRH antagonist degarelix was associated with lower rates of cardiovascular events. Although the included studies were limited to short follow-up periods (12 months), they may suggest a protective role for GnRH agonists in reducing the rate of new cardiovascular events. However, further studies with longer follow-up periods are required to confirm and validate these results [126,127].

A pooled analysis of two BAT trials involving 60 mCRPC patients showed favorable changes in body composition and lipid profile, as well as improved quality of life. After three cycles of BAT, 12.2% of patients experienced increased muscle mass, while their mean visceral and subcutaneous fat levels dropped by roughly 10 and 8%, respectively [128]. This is noteworthy since sarcopenia and high amounts of subcutaneous adipose tissue have been linked to a poor prognosis in mCRPC patients [129,130,131]. Furthermore, significant reductions in LDL and TG levels were observed after BAT treatment, with mean reductions of 12.4 and 26.9 mg/dL, respectively. After three BAT cycles, significant improvements in energy levels (*p* = 0.004) and physical functioning (*p* = 0.05) were observed, while the change in emotional well-being did not reach statistical significance [128]. Further investigation is necessary to ascertain whether these unique and promising findings will yield long-term health benefits for mCRPC patients.

BAT consistently outperformed ENZ regarding the quality of life (QoL), particularly regarding fatigue and sexual function, as documented in TRANSFORMER and RESOTRE trials [32,33]. Although BAT was associated with a lower incidence of fatigue, constitutional, and GI side effects than ENZ, it was associated with a higher incidence of edema, generalized pain, and sexual side effects [33].

## 8. Biomarker Selection

Since BAT provides clinical benefit to only about 30%-40% of patients, it is essential to identify biomarkers that can predict response and guide patient selection. During the TRANSFORMER study, blood samples were obtained and examined for the expression of full-length AR (AR-FL) and ARV-7. Neither of these biomarkers could predict the clinical outcome of patients treated with BAT [33]. Similarly, the RESTORE trial found no link between baseline circulating tumor cells and BAT response, as the difference in clinical outcomes between ARV-7-positive and -negative patients was not statistically significant (*p* = 0.13) [34]. Aside from this, in a retrospective study of 114 mCRPC patients across three BAT trials, 22 patients with a PSA reduction of 70% or greater were identified. Somatic DNA sequencing data were available for 15 of these responders, all of whom possessed inactivating mutations of TP53 and/or homologous recombination DNA repair genes [132]. However, as we mentioned earlier, the PSA50 and radiographic response rate of HRR-deficient patients enrolled in the BAT plus Olaparib trial was similar to that of HRR- intact patients, casting doubt on the efficacy of these mutations as predictive markers of response. In their search for a biomarker predicting response to BAT, Sena et al. used paired-matched biopsies of the soft tissue metastasis obtained from 24 participants in the COMBAT-CRPC study before and after 3 cycles of BAT. Ten of these twenty-four patients were responders, with a PSA50 response or tumor shrinkage of at least 30%. The abundance of AR mRNA and AR protein (both cytoplasmic and nuclear) in pre-BAT samples was similar between responders and non-responders. The authors generated a score for AR activity using a Mann–Whitney ranking (ARAMW) of the expression of 10 canonical AR target genes (KLK2, KLK3, FKBP5, STEAP1, STEAP2, PPAP2A, RAB3B, ACSL3, NKX3-1, TMPRSS2). Intriguingly, the pre-treatment ARAMW score was significantly higher in BAT responders compared to non-responders (*p* = 0.011). Using a cutoff point of 0.6, patients with a high ARAMW score (>0.6) exhibited better PSA responses (*p* = 0.01), more significant tumor shrinkage (*p* = 0.05), longer overall survival (*p* = 0.002), and a trend toward prolonged radiographic progression-free survival (*p* = 0.068). Such promising results suggest that the ARAMW score may have the potential to predict response to BAT prior to treatment, though this must be confirmed in future prospective studies. However, obtaining fresh tumor tissues is required in order to determine this biomarker, which seems difficult to apply in clinical practice; therefore, further studies are still warranted to identify reliable and applicable biomarkers for BAT. In this study, the baseline HRR gene mutation status failed to predict response to BAT [81].

In a recent study analyzing the responses of 41 mCRPC patients to BAT after progressing on ABI or ENZ, it was observed that patients who had received antiandrogen treatments were more likely to respond to BAT. Additionally, BAT responders exhibited a significantly longer duration of response to antiandrogens. Responders showed a trend toward having TP53 mutations on ctDNA analysis [133].

## 9. BAT and Molecular Imaging

**Figure 4:**
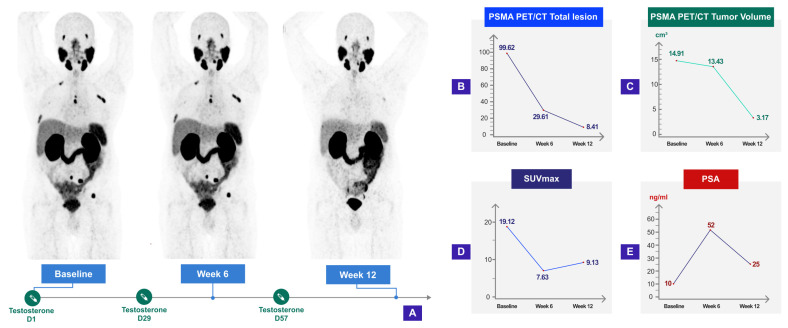
Serial PSMA PET/CT shows radiographic response despite overall PSA progression after three cycles of BAT. A 75-year-old patient with metastatic castration-resistant prostate cancer (mCRPC), who had been heavily pretreated with hormonal agents, was enrolled in the PSMA-BAT study (NCT04424654) and received 400 mg of intramuscular testosterone every 28 days for three cycles. The patient underwent PSMA-PET/CT imaging at baseline, at the six-week mark, and at the twelve-week mark. After three cycles of BAT, a clear radiographic response was observed despite an overall increase in PSA levels. Tumor volume, Total lesion, and SUVmax were reduced, while PSA levels more than doubled compared to baseline. (**A**) Timeline of testosterone injections, imaging acquisitions and images of the corresponding PSMA studies; Concurrent measurements are shown as (**B**) PSMA PET/CT total lesion chart; (**C**) PSMA PET/CT tumor volume chart; (**D**) SUVmax chart; (**E**) PSA chart.

Androgens regulate PSA expression at the gene level; testosterone administration directly stimulates PSA expression, potentially shortening the time to PSA progression of BAT studies. Therefore, it is worth considering clinical- or radiological-based endpoints as alternatives to PSA-based ones. Accordingly, discrepancies between PSA and radiographic response rates have been observed in BAT trials [34,35] (Figure 4). Utilizing novel molecular imaging modalities may aid researchers in addressing this issue, given that the emergence of these modalities has revolutionized the landscape of prostate cancer imaging. Particularly, prostate-specific membrane antigen (PSMA)-targeted imaging is gaining ground due to its improved detection rate, sensitivity, and specificity compared to conventional modalities for intermediate-to-high-risk PC [134,135]. PSMA is a transmembrane protein primarily expressed in the prostate gland, and its expression is upregulated up to 1000 fold in PC. This makes PSMA an ideal target for small-molecule radiotracers, including 68-Ga (68Ga-PSMA-11) and 18-F (18-FDCFPyL). Perhaps the superior diagnostic accuracy of PSMA PET/CT creates a potential role for this modality as an optimal biomarker for assessing treatment responses and survival outcomes. However, the impact of systemic treatments on PSMA expression, which is a relatively less-studied area, should not be overlooked.

The interplay between the androgen-signaling-axis and PSMA expression is rather complex, and the current body of evidence has yielded heterogeneous results. Initial preclinical studies suggested an inverse association between the androgen signaling pathway and PSMA expression: while androgens suppressed PSMA expression in AR-positive PC cell lines, ADT and NHAs upregulated PSMA expression in both castration-sensitive and -resistant cells [136,137,138]. In clinical settings, however, the results are even more inconsistent. Some studies documented PSMA upregulation after commencing ADT or NHAs, whereas others reported dichotomous results, suggesting that the effects of androgen-targeted treatments on PSMA may depend on the duration of therapy and the hormonal sensitivity state of the tumor [139,140,141,142]. Furthermore, substantial intertumoral (between different metastatic sites) and intratumoral (between different cores of a metastasis) heterogeneity has been observed, which may reflect different cell responses to the same therapies within a patient and/or a metastasis site [143]. In contrast to initial studies, a recent study reported that AR-negative tumors tend to exhibit low-to-absent levels of PSMA, implying that AR signaling might be required for FOLH1/PSMA expression. They also demonstrated that the difference between PSMA-positive and -negative tumor samples regarding AR binding to the FOLH1 locus and AR activity was insignificant. These results suggest that although AR signaling modulates FOLH1/PSMA expression, other mechanisms may contribute to the profound changes observed regarding PSMA expression in CRPC, given that reduced or lost AR signaling did not result in FOLH1 silencing [143]. Overall, it appears that short-term ADT (<6 weeks) might upregulate PSMA expression, while receiving ADT for longer terms (>12 weeks) leads to decreased levels of PSMA expression, perhaps due to both downregulation and cell death [144]. In spite of this heterogeneity, none of the studies that evaluated PSMA expression after the long-term (>3 months) administration of androgen-targeting drugs (either ADT or NHAs) have reported PSMA flares or upregulations, implying that such events are temporary and resolving [145].

Although PSMA PET/CT has been previously used in tandem with other systemic treatments to evaluate the response and predict OS in patients with metastatic PC, the effects of BAT as a novel systemic treatment on PSMA expression remain relatively unexplored. Until now, only one study has implemented PSMA/PET imaging to evaluate BAT: Six mCRPC patients underwent 18-FDCFPyL PET/CT before and after three cycles. Two-thirds of the patients achieved PSA50, and one demonstrated an objective response on conventional imaging. While conventional imaging revealed no progression after three BAT cycles, 18F-DCFPyL PET/CT detected progression in half of the patients (3/6). After three months, these patients exhibited progression on conventional imaging as well. On the other hand, patients who did not progress on 18-FDCFPyL PET/CT after three cycles of BAT remained free of radiographic progression for 9–12 months. Although these preliminary results might suggest that PSMA-based imaging may be more favorable in order to detect early progression in BAT, further prospective studies are required to validate these findings.

Systemic treatments may alter PSMA expression on multiple accounts. Firstly, PSMA is thought to direct cellular growth and promote tumor progression by acting on glutamate receptors and activating the pro-survival protein kinase B (AKT)/phosphatidylinositol 3-kinase pathways [135]. As an apoptotic mechanism, BAT may therefore downregulate PSMA at the protein level, resulting in tumor regression. Secondly, testosterone-induced rearrangements of AR may cause downregulation in PSMA expression while the tumor maintains its viability. Alternatively, the flare phenomenon could have a role, which may create an interpretation dilemma in the setting of BAT, given the rise of PSA that is observed in a subset of patients.

Comprehending PSMA expression changes following treatments, such as BAT, may enhance the diagnostic performance of PSMA-based modalities. Therefore, serial PSMA PET studies that compare baseline characteristics with follow-up scans are necessary to provide us with the much-required data regarding the impact of BAT on PSMA expression and the role of PSMA-based imaging in response assessment. Currently, only one ongoing study aims to evaluate the kinetics of ^68^Ga-PSMA-11/PET maximal standard uptake value (SUVmax) following three cycles of BAT (NCT04704505). The primary endpoint of this biomarker study is to evaluate the correlation between baseline Galium68-PSMA/PET SUVmax and response to BAT in mCRPC patients.

## 10. Conclusions

Although physiological levels of androgens may fuel the fire of prostate cancer, supraphysiological androgens have shown the capacity to extinguish it. The current body of evidence suggests there are multiple parallel mechanisms mediating this paradoxical growth suppression. While significant progress has been made in understanding the contributing pathways for this paradox, further studies are still needed to extend our knowledge of the underlying mechanisms. This, in turn, enables us to explore new therapeutic horizons and pinpoint more effective combination therapies in the fight against CRPC. Bipolar androgen therapy has produced a sustained PSA or objective response in 30–40% of mCRPC patients. BAT is relatively safe and extremely well-tolerated, and it has shown improvements in patients’ quality of life, particularly in terms of fatigue and sexual function. Furthermore, even short durations of BAT have exhibited the potential to restore sensitivity to antiandrogens such as enzalutamide, allowing for an extended duration of current androgen ablative therapies before resorting to more cytotoxic treatments like chemotherapy. While phase 3 clinical trials are still pending, further studies are required to identify biomarkers capable of predicting response and to find optimal sequencing and combinations that enhance BAT’s efficacy and prolong the duration of response. We eagerly await the results of ongoing studies, hoping they facilitate the integration of BAT into the standard of care for advanced prostate cancer patients, which may translate into more favorable outcomes for these patients.

## Figures and Tables

**Table 1:** Completed clinical trials evaluating the efficacy of BAT in prostate cancer.

Study Name	Setting	Number of Patients	Design	Regimen	PSA 50	ORR	rPFS	crPFS	PSAPFS
Pilot	CRPC with low to moderate metastasis burden	16	Single arm, pilot study	BAT + Oral etoposide	4/14 (28.6%)	5/10 (50%)	NR	NR	NR
BATMAN	Low volume mHSPC or nmHSPC	29	Single arm, Phase 2	ADT followed by BAT-ADT alternation	PSA < 4: 17/29 (58.6%)	8/10 (80%)	NR	NR	NR
RESTORE	Cohort A: mCRPC post-ENZ	30	Multi-cohort, Single center, phase2	BAT	9/30 (30%)	6/12 (50%)	NR	8.6 months	3.3 months
	Cohort B: mCRPC post-ABI	29		BAT	5/29 (17.2%)	2/7 (28.5%)	5 months	4.3 months	NR
	Cohort C: CRPC (no prior NHA exposure)	29		BAT	4/29 (13.7%)	4/13 (30.7%)	NR	8.5 months	1 month
TRANSFORMER	mCRPC post-ABI	195	Multicenter, Randomized, Phase 2	BAT or ENZ	24/85 (28.2%) vs. 24/94 (25.5%)	8/33 (28.2%) vs. 1/24 (4.2%)	6.05 vs. 8.29 months	5.6 vs. 5.7 months	2.8 vs. 3.8 months
COMBAT	mCRPC post-ENZ/ABI ± Taxane chemo	45	Single arm, multi center, Phase 2	BAT followed by BAT+Nivolumab	18/45 (40%)	10/42 (23.8%)	5.7 months	NR	NR
BAT plus Olaparib	mCRPC post-ENZ and/or ABI	36	Single arm. Single center, phase 2	BAT + Olaparib (300 mg P.O BID)	16/36 (44%)	7/13 (54.5%)	NR	13 months	7 months

BAT, Bipolar Androgen Therapy; PSA50, PSA decline ≥ 50; ORR, Objective Response Rate; rPFS, radiographic Progression-Free Survival; crPFS, clinical or radiographic Progression-Free Survival; PSA PFS, PSA Progression-Free Survival; CRPC, Castration-Resistant Prostate Cancer; NR, not reported; nmCRPC, non-metastatic Castration-Resistant Prostate Cancer; ADT, Androgen Deprivation Therapy; mCRPC, metastatic Castration-Resistant Prostate Cancer; ENZ, Enzalutamide; ABI, Abiraterone; NHA, Next-generation hormonal agents.

**Table 2:** Ongoing clinical trials evaluating BAT.

Study	Protocol	Number of pts	Patient Population	Design	Primary Endpoint	CTC Identifier
BAT-RAD	BAT + Radium-223	47	mCRPC	Single arm, Phase 2	Median rPFS	NCT04704505
HiTeCH	BAT + Carboplatin	30	mCRPC with confirmed HRR-mutations	Single arm, Phase 2	PSA response rate	NCT035522064
PSMA-BAT	BAT	20	mCRPC post-ABI/ENZ	Pilot, Single arm, Biomarker study	Ga-68-PSMA uptake and response to BAT	NCT04424654
Ex-BAT	BAT + Darolutamide	47	mCRPC post-ABI	Single arm, Multi center, Phase 2	rPFS rate	NCT04558866
STEP-UP	Arm A: Continuous ENZ	50	mCRPC post-ABI ± ADT	Three-arm randomized, Phase 2	crPFS	NCT04363164
	Arm B: Fixed cycles of Sequential BAT and ENZ	50				
	Variable cycles of sequential BAT and ENZ	50				

BAT, Bipolar Androgen Therapy; CTC, Clinical Trial Code; mCRPC, metastatic Castration Resistant Prostate Cancer; rPFS, radiographic Progression-Free Survival; HRR, Homologous Recombination Repair, ABI, Abiraterone; ENZ, Enzalutamide; ADT, Androgen Deprivation Therapy, crPFS, clinical or radiographic Progression-Free Survival.

## Data Availability

No new data were created or analyzed in this study. Data sharing is not applicable to this article.
